# Characteristics of sports brand content on social media and their effects on consumers

**DOI:** 10.3389/fspor.2025.1721424

**Published:** 2025-11-21

**Authors:** Yue Liu

**Affiliations:** School of Physical Education, Heze Medical College, Shandong, China

**Keywords:** social media, sports brand content, brand attitude, brand loyalty, purchase intention

## Abstract

**Introduction:**

With the rise of short-video platforms, social media—especially TikTok—is profoundly transforming the marketing model of the sports industry. These platforms provide brands with targeted consumer engagement channels that enhance communication efficiency and drive digitalization, creating a need to understand the specific mechanisms through which content influences consumer behavior.

**Methods:**

This study empirically investigated these mechanisms by surveying 1,400 adult consumers in mainland China who have purchased sports products on TikTok. Data were collected using validated scales for brand content characteristics (informativeness, entertainment, interactivity, authenticity), brand attitude, brand loyalty, and purchase intention. The proposed relationships were analyzed using structural equation modeling with SPSS 28.0 and AMOS 28.0.

**Results:**

The findings reveal that all four content characteristics significantly impact purchase intention. Notably, the effects of informativeness and interactivity were found to be comparable in magnitude. Furthermore, brand attitude and brand loyalty served as significant mediators in the relationship between content characteristics and purchase intention. Crucially, the indirect effects through these psychological mediators surpassed the direct influence of the content itself.

**Discussion:**

The results underscore that the primary value of TikTok content marketing lies not in direct persuasion but in building brand assets. We conclude that sports brands can optimize their digital strategy by prioritizing content that delivers valuable information and fosters active interaction, thereby directly and indirectly enhancing purchase intention through the cultivation of positive brand attitude and steadfast brand loyalty.

## 1 Background

With the widespread adoption of the internet, social media has become an integral part of daily life. The rise of short-form video content has fundamentally transformed users' media consumption habits, driving a paradigm shift in marketing strategies (1). As one of the most influential short-video social platforms globally, TikTok has consistently ranked as the most downloaded application worldwide since 2020 (2). Recognized as one of the most valuable global brands (3), TikTok leverages precise interest-based algorithms to enhance the dissemination of brand content significantly, optimizing engagement and audience reach (4).

In recent years, driven by the wave of digitalization, the sports market has experienced continuous expansion. Sports are not only a social and cultural activity but also a highly commercialized industry encompassing multiple sectors, including sports events, athletic apparel, and fitness services (5). With the rise of social media, the marketing strategies within the sports industry have shifted from traditional advertising and sponsorship models toward more interactive and personalized digital marketing approaches. Notably, the emergence of short-video platforms has provided sports brands with more precise consumer targeting channels, thereby enhancing brand communication efficiency and user engagement (6).

Social media marketing has become an essential component of the sports industry. Sports brands leverage TikTok for market promotion, using brand storytelling, product showcases, and sports scene videos to increase brand awareness and consumer engagement (7). For example, global brands such as Nike and Adidas have established a presence on TikTok, utilizing KOL (Key Opinion Leader) marketing, branded hashtag challenges, and interactive campaigns to expand their brand influence (8). TikTok's short-video format not only strengthens the visual representation of brands but also enhances brand recognition and loyalty through interactive mechanisms. In social media marketing, brand content characteristics play a crucial role. Informativeness improves consumers' brand awareness and trust (9), while authenticity enhances brand authenticity and reduces consumer uncertainty (10). Interactivity fosters engagement between brands and consumers, increasing brand stickiness (11), whereas entertainment value enhances brand appeal and user participation (12). On the TikTok platform, brands employ diversified content strategies, such as short videos, live streaming, and branded challenges, to shape their brand image and create immersive experiences that influence consumer brand attitudes and purchase intentions (13). Overall, in the highly commercialized sports industry, TikTok provides brands with precise audience targeting, enabling them to enhance consumer brand perception through diversified content strategies. Additionally, brand content characteristics reduce consumer uncertainty regarding brand perception, enhance brand appeal and user engagement, and ultimately influence consumer attitudes and purchase intentions (14).

Therefore, This study employed rigorous screening criteria to establish a validated cohort of 1,400 consumers with documented sports product purchase histories on TikTok(Douyin). This methodological approach ensures the ecological validity and practical relevance of our findings to Douyin's e-commerce ecosystem. We constructed and empirically validated a dual-mediation model examining “content characteristics → brand attitude/brand loyalty → purchase intention.” This sophisticated analytical framework provides superior explanatory power compared to single-mediation models by revealing the complex mechanisms through which short-video content influences consumer behavior, while distinguishing between immediate affective responses and long-term relationship building. The research innovatively integrates the classical Stimulus-Organism-Response (S-O-R) framework with TikTok unique platform affordances, including algorithmic recommendation systems and live-streaming interactive features. This theoretical advancement offers a more contextually appropriate explanatory model for short-video marketing dynamics. Examining the relationship and underlying mechanisms between sports brand content characteristics on the TikTok platform and consumer purchasing behavior holds significant theoretical and practical implications for understanding and optimizing digital marketing strategies in the sports industry.

**Figure 1:**
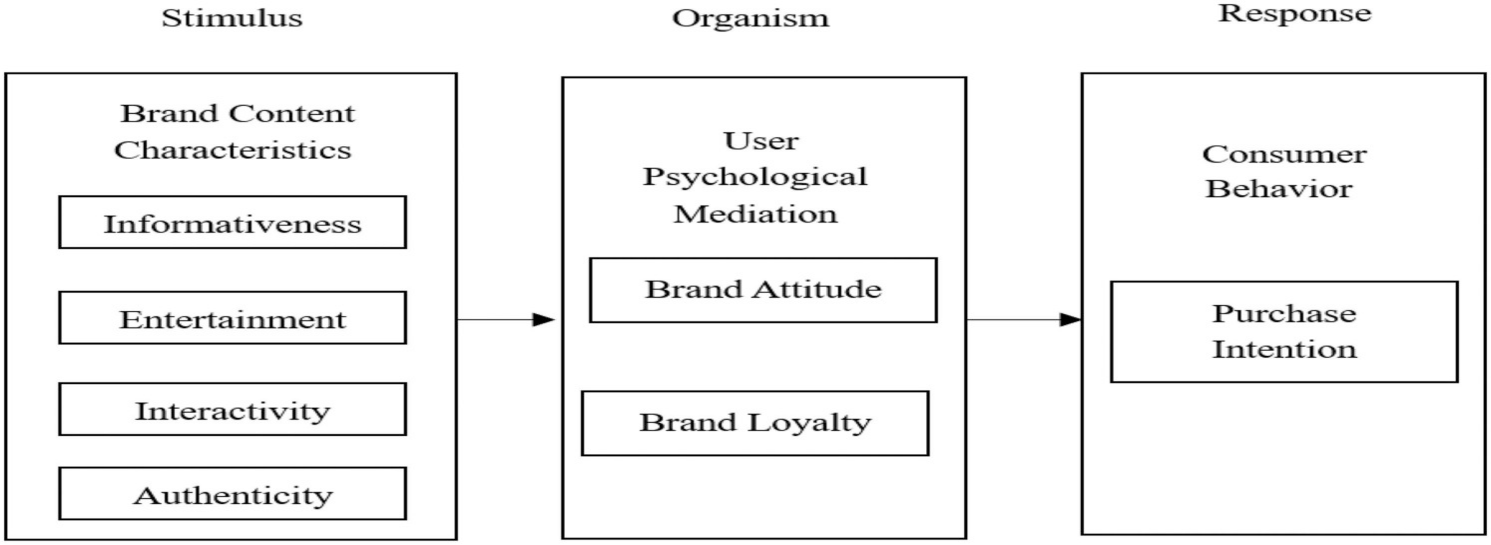
S-O-R theoretical model diagram.

The relationship between brand content characteristics and purchase intention has been widely explored in academic research, particularly within the social media marketing context (15). From a theoretical perspective, the Stimulus-Organism-Response (S-O-R) model posits that external stimuli (i.e., brand content characteristics) first influence an individual's cognitive and emotional state, which subsequently determines their behavioral responses (16). This model framework elucidates the core theoretical logic of our investigation. The distinctive affordances of the TikTok platform—specifically its algorithmic recommendation systems, live-streaming capabilities, and short-form video architecture—serve as carriers and shapers of specific brand content attributes (informativeness, entertainment, interactivity, authenticity). These content attributes function as “ Stimulus” (S) that collectively influence users' internal psychological “Organism”(O)—manifested through brand attitude formation and brand loyalty development—ultimately driving their behavioral “Response”(R) in the form of purchase intention (Figure 1). In the realm of social media marketing, brand content characteristics significantly impact consumers’ psychological and emotional processes, ultimately shaping their purchase decisions (17). Specifically, informativeness reduces consumers’ perceived risk in the decision-making process, while entertainment value enhances their positive emotional experiences. Interactivity fosters consumers’ trust and reliance on the brand, which in turn promotes positive purchasing behaviors (18). Additionally, word-of-mouth (WOM) effects facilitate consumer opinion exchange and social interactions, reinforcing brand social identity and authenticity, reducing uncertainty and hesitation in decision-making, and significantly increasing consumers’ adoption of brand marketing information, thereby strengthening their purchase intention (14). Based on the above analysis, it can be inferred that the content characteristics of sports brands on TikTok effectively stimulate consumers’ cognitive and emotional experiences, thereby positively influencing their purchase intentions. Accordingly, the following research hypothesis is proposed: H1: The content characteristics of sports brands on TikTok have a significant positive impact on purchase intention.

Brand attitude represents consumers' overall evaluation of a brand, encompassing cognitive attitude (based on informational judgment) and affective attitude (derived from emotional responses to brand experiences) (19). Within the social media environment, the informativeness and authenticity of brand content are key factors influencing cognitive brand attitudes. According to the Information Adoption Model (IAM), consumers' acceptance of brand content primarily depends on information quality and authenticity (20). High-quality information reduces consumers' cognitive uncertainty regarding a brand, helping establish a professional and credible brand image, which subsequently enhances cognitive brand attitudes (21). Meanwhile, authenticity, as a core value of brand information, further strengthens consumers' trust in the brand, fostering positive cognitive attitudes (22). Additionally, interactive experiences and emotional resonance derived from brand content stimulate consumers' affective responses, thereby reinforcing affective brand attitudes (23). According to the Theory of Reasoned Action (TRA), an individual's behavioral intention is determined by attitude and subjective norms. In the brand marketing domain, brand attitude reflects consumers' cognitive evaluation and emotional inclination toward a brand, serving as a crucial indicator of brand relationship quality (24). Consumers with positive brand attitudes tend to exhibit higher brand trust and stronger emotional connections, which, in turn, significantly enhance their purchase intention. Based on the above theoretical framework and literature review, it can be inferred that sports brands can strengthen consumers' cognitive brand attitudes by providing high-quality, credible, and authentic content. Simultaneously, brand interactivity and emotional engagement further enhance consumers' affective brand attitudes, forming a comprehensive and positive brand perception, which subsequently drives purchase intention. Accordingly, the following hypothesis is proposed: H2: Brand attitude mediates the relationship between the content characteristics of sports brands on TikTok and purchase intention.

In the social media environment, brand content characteristics play a critical role in shaping brand loyalty. According to Social Exchange Theory, the interaction between consumers and brands is perceived as a value exchange process. Prior research has identified that social media marketing activities, through key dimensions such as trendiness, personalization, and word-of-mouth (WOM) effects, significantly influence brand equity and brand loyalty (25). Furthermore, the effectiveness of social media marketing is reflected in the substantial enhancement of brand loyalty (26). Therefore, high-quality content engagement serves as a crucial driver in strengthening brand loyalty. Brand loyalty is widely defined as a consumer's sustained preference and repeated purchase behavior toward a specific brand (27). According to the Theory of Reasoned Action (TRA) and the Attitude-Behavior Loyalty Model, loyal consumers not only demonstrate consistent emotional and cognitive preferences but also exhibit clear behavioral intentions, meaning they are more likely to continue purchasing products or services from a specific brand (28). Empirical studies further confirm that brand loyalty is one of the core determinants of consumers' repeat purchase intention (29). Based on this, it can be inferred that sports brands can enhance consumer brand identification and engagement through effective content management and interactive strategies, thereby fostering long-term, stable brand loyalty. Given that brand loyalty is a key determinant of repeat purchase intention, this study proposes the following hypothesis: H3: Brand loyalty mediates the relationship between the content characteristics of sports brands on TikTok and purchase intention.

**Figure 2:**
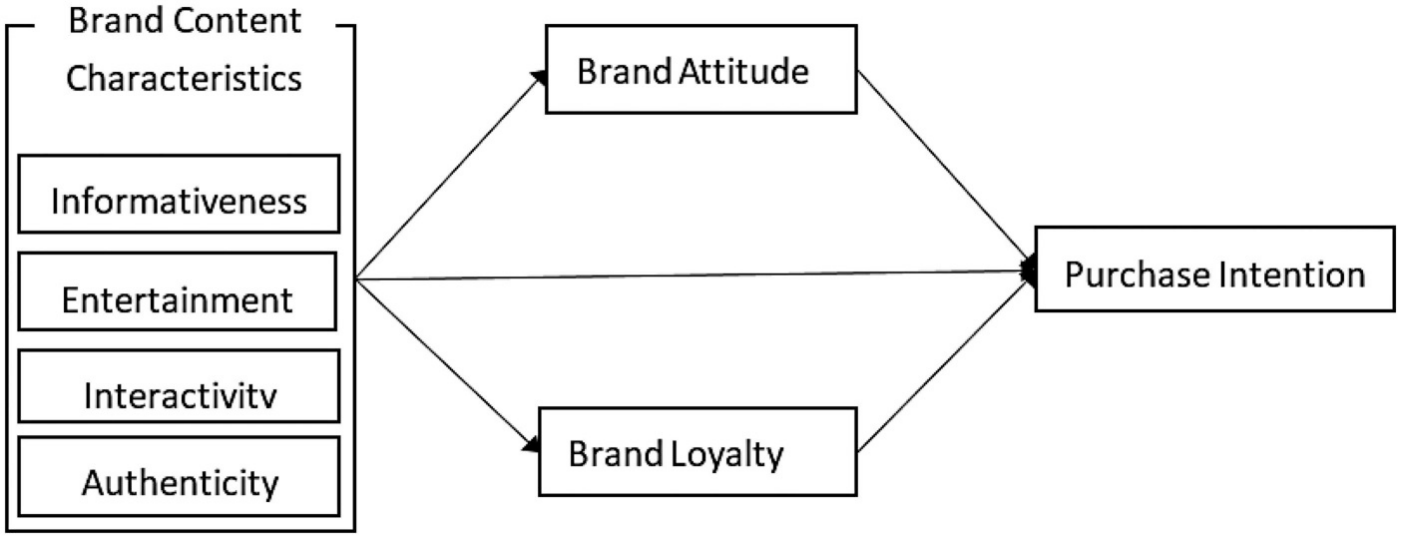
Hypothetical model diagram.

This study examines the impact of sports brand content characteristics on consumer behavior in the social media context. The proposed theoretical model suggests that brand content characteristics indirectly influence consumer purchase intention through the mediating roles of brand attitude and brand loyalty, thereby revealing the psychological mechanisms underlying the relationship between brand content and consumer purchasing behavior. The findings derived from this model provide both theoretical guidance and practical implications for sports brands to optimize their social media marketing strategies, enhance consumer engagement, and effectively drive purchasing behavior (Figure 2).

## 2 Method

### 2.1 Survey objects

This study reviewed and consolidated existing research scales related to sports brand content characteristics, brand attitude, brand loyalty, and purchase intention on TikTok(Douyin). After a rigorous screening and refinement process, a customized scale was developed to suit the objectives of this study. To ensure the validity of the research instrument, expert opinions from the sports industry and feedback from TikTok users were collected, leading to the development of a preliminary survey (pilot study). Informed consent was issued and ethical approval was obtained from Myongji University. The pilot survey was conducted between March 10 and April 10, 2024, with a sample consisting of consumers who had previously purchased sports products via the TikTok platform in Mainland China. A total of 200 online questionnaires were distributed via clickable survey links, and 135 responses were collected. Based on the survey results, necessary modifications and refinements were made, leading to the finalization of the survey instrument. The study employed a non-probability sampling method, specifically a purposive sampling technique, to target consumers from economically developed cities in Southern China (Shanghai, Nanjing) and Northern China (Beijing, Jinan). This approach was adopted to efficiently reach the core user base of TikTok e-commerce for sports products. The final version of the questionnaire was distributed through an online survey platform. A total of 1,500 questionnaires were sent to consumers from economically developed cities in Southern China (Shanghai, Nanjing) and Northern China (Beijing, Jinan) between July 10 and August 14, 2024. Respondents were required to complete the survey via an online survey link, and response data were monitored in real time through the survey platform's backend management system. Incomplete or blank responses were considered invalid. Among the returned responses, 100 surveys were excluded due to issues such as dishonest answers, blank responses, and duplicate submissions. Ultimately, 1,400 valid responses from TikTok users who had purchased sports products were analyzed. Sample Characteristics. The demographic distribution of the survey participants is as follows (detailed in Table 1): Gender: Male (758, 54.1%), Female (642, 45.9%); Age Distribution: Below 18 years old (92, 6.6%), 19–25 years old (574, 41.0%), 26–30 years old (483, 34.5%), 31–49 years old (221, 15.8%), 50 years and above (30, 2.1%); Educational Background: Below high school (54, 3.9%), High school graduate (175, 12.5%), University graduate (1,098, 78.4%), Postgraduate and above (73, 5.2%); Occupational Distribution: Government employees (112, 8.0%), Professionals and technical personnel (342, 24.4%), Corporate employees (306, 21.9%), Service industry personnel (192, 13.7%), Industrial workers (150, 10.7%), Students (198, 14.1%), Self-employed individuals (92, 6.6%), Unemployed individuals (8, 0.6%); Monthly Income: Below 3,000 RMB (116, 8.3%), 3,001–6,000 RMB (807, 57.6%), 6,001–10,000 RMB (397, 28.4%), Above 10,001 RMB (80, 5.7%); Average Daily TikTok Viewing Time: 1–2 h (818, 58.4%), 2–3 h (465, 33.2%), More than 3 h (117, 8.4%); Brand Preferences on TikTok, Among official TikTok sports brand accounts, the most-followed brands among respondents are: Nike (246, 17.6%), Adidas (244, 17.4%), Puma (170, 12.1%), Under Armour (75, 5.4%), New Balance (186, 13.3%), Skechers (76, 5.4%), Anta (250, 17.9%), Reebok (35, 2.5%), Asics (60, 4.3%), Other brands (58). This distribution suggests a well-balanced representation of consumer preferences in the dataset. We have performed further analyses to see if there are any significant moderating effects of demographic variables (e.g., age, gender). However, we couldn't find any significant effect. The detailed demographic statistics are presented in Table 1.

**Table 1:** Profile of the sample.

Demographics	Category	Number of people	Percentage (%)
Gender	Male	758	54.1
	Female	642	45.9
Age	Under 18 years of age	92	6.6
	19–25	574	41
	26–30	483	34.5
	31–49	221	15.8
	Over 50 years old	30	2.1
Education level	Below High School	54	3.9
	High School Graduate	175	12.5
	University Graduate	1098	78.4
	Postgraduate and Above	73	5.2
Monthly income	Below 3,000 RMB	116	8.3
	3,001–6,000 RMB	807	57.6
	6,001–10,000 RMB	397	28.4
	Above 10,001 RMB	80	5.7
Average duration	1–2 h	818	58.4
	2–3 h	465	33.2
	More than 3 h	117	8.4
Most followed sports brands on TikTok	Nike	246	17.6
	Adidas	244	17.4
	Puma	170	12.1
	Under Armour	75	5.4
	New Balance	186	13.3
	Skechers	76	5.4
	Anta	250	17.9
	Reebok	35	2.5
	Asics	60	4.3
	Others	58	4.1

### 2.2 Measurement tools

The questionnaire for this study was developed based on prior research and constructed using a Likert scale (1 = Strongly Disagree, 3 = Neutral, 5 = Strongly Agree). The survey consisted of the following components: 24 items related to TikTok sports brand content characteristics, 6 items measuring brand attitude, 8 items assessing brand loyalty, 3 items evaluating purchase intention. Additionally, seven demographic questions were included, covering gender, age, education level, occupation, monthly income, daily time spent on TikTok, and preferred sports brands. In total, 48 items were designed to validate the research model (Table 2).

**Table 2:** Used to measure the relationship between sports brand content information and brand attitude, brand loyalty, and purchase intention.

Variables	Questions	Sources
Informativeness	The sports brand content on TikTok provides the latest information.	Çil et al. (30), Kwon et al. (31)
	The sports brand content on TikTok delivers accurate information.	
	The sports brand content on TikTok offers important information.	
	The sports brand content on TikTok makes it easy to access product-related information.	
	The sports brand content on TikTok helps you make the best decisions.	
	The sports brand content on TikTok provides high-quality information.	
Entertainment	The sports brand content on TikTok is lively and interesting.	
	The sports brand content on TikTok is exciting.	
	The sports brand content on TikTok is enjoyable.	
	The sports brand content on TikTok is visually appealing.	
	The sports brand content on TikTok creates a pleasant mood.	
	The sports brand content on TikTok feels fresh and captures my interest.	
Interactivity	The sports brand content on TikTok allows me to interact with like-minded individuals.	
	The sports brand content on TikTok enables me to exchange ideas with others.	
	The products or services presented in the sports brand content on TikTok are convenient to discuss.	
	The products or services presented in the sports brand content on TikTok are easy to communicate about.	
	The products or services provided in the sports brand content on TikTok can be accessed anytime, anywhere.	
	The products or services presented in the sports brand content on TikTok receive timely feedback.	
Authenticity	The sports brand content on TikTok feels authentic.	
	The sports brand content on TikTok is trustworthy.	
	The sports brand content on TikTok is objective.	
	The sports brand content on TikTok is highly appealing.	
	The sports brand content on TikTok contains essential information.	
	The sports brand content on TikTok meets my expectations.	
Cognitive attitude	I think this sports brand is excellent.	Kwon et al. (31)
	I believe this sports brand offers good value for money.	
	I find this sports brand satisfactory.	
Affective attitude	I have a stronger preference for this sports brand.	
	I trust this sports brand more.	
	Among similar sports brands, I have a stronger interest in this one.	
Attitudinal loyalty	I am very loyal to this sports brand.	Dapena-Baron et al. (32)
	This sports brand is my first choice.	
	I actively search for information related to this sports brand.	
	If needed, I will prioritize this sports brand.	
Behavioral loyalty	This sports brand is exactly what I need.	
	If needed, I will make sure to purchase this sports brand's products.	
	I have recommended this sports brand to others.	
	I let others know that I use this sports brand.	
Purchase intention	I intend to purchase this sports brand in the future.	Kwon et al. (31)
	If I want to buy a similar type of sports brand, I am highly likely to choose this one.	
	Even if this sports brand is priced higher than other similar sports brands, I am still highly likely to purchase it.	

#### 2.2.1 Brand content characteristics

In this study, brand content is defined as short videos either directly created and produced by enterprises or co-created in collaboration with content producers. Brand content integrates both brand information and product-related content, aiming to measure consumers’ perceptions of sports brand content while aligning with the characteristics of TikTok. The key dimensions of TikTok brand content characteristics include informativeness, entertainment, interactivity, and authenticity. The survey items were adapted and modified from the study by Çil et al. (30) to better fit the context of this research (30) Kwon et al. (31). A total of 24 items were used to measure brand content characteristics, distributed as follows: 6 items measuring informativeness, 6 items measuring entertainment, 6 items measuring interactivity, 6 items measuring authenticity. All items were measured using a 5-point Likert scale (1 = Strongly Disagree, 5 = Strongly Agree).

#### 2.2.2 Brand attitude

Early research on brand attitude primarily focused on a single-dimensional perspective. However, consumer brand attitude is a key predictor of both purchase intention and brand choice. Brand attitude consists of two core components: cognition and affect—where cognition is formed through beliefs, cognition triggers emotions, and emotions lead to actions. The survey items used in this study were adapted and modified from Kwon et al. to better align with the research context (31). A total of six items were used to measure brand attitude, including: Three items assessing cognitive brand attitude, Three items assessing affective brand attitude. All items were measured using a 5-point Likert scale (1 = Strongly Disagree, 5 = Strongly Agree).

#### 2.2.3 Brand loyalty

In consumer-brand relationship research, brand loyalty is a fundamental concept. Brand loyalty refers to the degree to which customers repeatedly purchase or use a specific brand's products or services (33). Extensive research in marketing literature has explored various dimensions of consumer brand loyalty. The survey items used in this study were adapted and modified from Dapena-Baron et al. to better fit the research context (32). A total of eight items were used to measure brand loyalty, divided into two key dimensions: Four items measuring attitudinal loyalty, Four items measuring behavioral loyalty. All items were measured using a 5-point Likert scale (1 = Strongly Disagree, 5 = Strongly Agree).

#### 2.2.4 Purchase intention

Purchase intention is regarded as an individual's motivational inclination to engage in a specific purchasing behavior. While purchase intention does not equate to actual purchasing behavior, it plays a crucial role in guiding consumer decision-making and actions. The survey items used in this study were adapted and modified from Kwon et al. (31). The measurement consisted of three items assessing purchase intention. All items were measured using a 5-point Likert scale (1 = Strongly Disagree, 5 = Strongly Agree).

### 2.3 Reliability test

Cronbach's *α* coefficient is used to measure internal consistency reliability of the scale, with values ranging from 0 to 1, where higher values indicate greater reliability: *α* ≥ 0.9: Excellent internal consistency, 0.8 ≤ *α* < 0.9: Good reliability, 0.7 ≤ *α* < 0.8: Acceptable reliability, *α* < 0.7: Insufficient reliability, generally considered inadequate for research use. All items were measured using a 5-point Likert scale (Table 3). Based on previous studies, 24 items were used to measure the four characteristics of social media content: Informativeness (*α* = 0.875), Entertainment (*α* = 0.868), Interactivity (*α* = 0.905), Authenticity (*α* = 0.887).Brand attitude was assessed using six items adapted from Kwon et al. (31), with reliability coefficients of: Cognitive attitude (*α* = 0.812), Affective attitude (*α* = 0.831). Brand loyalty was measured using eight items adapted from Dapena-Baron et al. (32), and Çil et al. (30). The two dimensions of brand loyalty had the following reliability scores: Attitudinal loyalty (*α* = 0.860), Behavioral loyalty (*α* = 0.835).Purchase intention was measured using three items from Kwon et al. (31), with a reliability coefficient of *α* = 0.844.

**Table 3:** Summary of Cronbach's *α* authenticity analysis for social media characteristics, brand attitude, brand loyalty, and purchase intention.

Variables	Questions	Sources	Cronbach's *α*
Informativeness	6	Çil et al. (30), Kwon et al. (31)	0.878
Entertainment	6		0.868
Interactivity	6		0.905
Authenticity	6		0.887
Cognitive attitude	3	Kwon et al. (31)	0.812
Affective attitude	3		0.831
Attitudinal loyalty Behavioral loyalty	4 4	Dapena-Baron et al. (32)	0.86
			0.835
Purchase intention	3	Kwon et al. (31)	0.844

### 2.4 Mathematical and statistical methods

This study utilized SPSS 28.0 and AMOS 28.0 software for data processing and analysis. The valid dataset was imported into SPSS 28.0, where reliability analysis, exploratory factor analysis (EFA), and confirmatory factor analysis (CFA) were conducted to examine the reliability and validity of the data. AMOS 28.0 was used to validate the structural validity of the measurement scales. Additionally, correlation analysis and regression analysis were performed to assess relationships among variables. The statistical significance threshold was set at *p* < 0.05. The study aims to explore whether sports brand content characteristics significantly impact purchase intention, while also uncovering the causal relationships among different variables.

## 3 Results and analyses

### 3.1 Common method bias test

To control for potential confounding effects caused by artificial covariation and to prevent misleading research conclusions, this study conducted Harman's Single-Factor Test before performing data analysis to assess the presence of common method bias (CMB). According to Podsakoff et al. (34), if the variance explained by the first unrotated principal component is less than 50%, common method bias is not considered a significant issue (34). To mitigate common method bias, we implemented both procedural and statistical remedies. Procedurally, we randomized the sequence of measurement items for brand content characteristics, brand attitude, brand loyalty, and purchase intention to disrupt response patterns, and strategically incorporated reverse-scored items to activate cognitive processing and detect insufficient effort responding. Statistically, we conducted a latent method factor analysis. While non-negligible, the changes in model fit indices fell below the threshold for substantive concern (*Δ*CFI/*Δ*TLI < 0.05). This suggests that while common method variance is present, it does not substantially alter the interpretation of the theoretical relationships. In this study, all measurement items across variables were subjected to Harman's Single-Factor Test. As shown in Table 4, the variance explained by the first extracted factor was 35.553%, which is well below the 50% threshold, indicating that common method bias is not a serious concern in this study's measurement scales.

**Table 4:** Common method bias test.

Igredients	Component initial eigenvalues		
	Total	Variance %	Cumulative %
1	14.577	35.553	35.553
2	2.725	6.647	42.199
3	2.269	5.533	47.733
4	1.881	4.588	52.321
5	1.816	4.43	56.751
6	1.691	4.124	60.874
7	1.149	2.803	63.678
8	1.136	2.77	66.447

### 3.2 Correlation analysis among sports brand content characteristics, brand attitude, and brand loyalty

Correlation analysis examines the relationship between variables by calculating their correlation coefficients. In social science research, Pearson's correlation coefficient is one of the most commonly used statistical measures to assess the strength and direction of associations between variables. If the correlation coefficient passes the significance test, it indicates a statistically significant positive or negative correlation between the variables. Conversely, if the correlation coefficient does not pass the significance test, it suggests that no statistically significant relationship exists between the variables (35).

To examine whether there are correlations among TikTok sports brand content characteristics, brand attitude, brand loyalty, and purchase intention, correlation analysis was conducted. If significant correlations exist, further model construction can be employed to determine the precise relationships between these variables. To ensure the discriminant validity of the constructs, this study adopted the Average Variance Extracted (AVE) method, a rigorous approach commonly used in empirical research. According to the AVE criterion, the square root of AVE for each factor should be greater than the correlation coefficients between that factor and any other variables, indicating adequate discriminant validity. As shown in Table 5, the square root of AVE for each construct is greater than the standardized correlation coefficients between constructs, confirming that the measurement model exhibits sufficient discriminant validity. Additionally, the lower triangular matrix presents the Pearson correlation coefficients, and the results indicate that all variables are significantly positively correlated (*P* < 0.01, Pearson correlation coefficient > 0). Furthermore, the AVE square root values for all constructs exceed their respective inter-variable correlations, demonstrating that the measurement scale possesses strong discriminant validity. As shown in Table 6, The Heterotrait-Monotrait (HTMT) ratio, which calculates the mean of indicator correlations across constructs relative to the mean of correlations within the same construct, should maintain values below 0.85 to establish discriminant validity. Our analysis demonstrates that all HTMT values in this study fall below this conservative threshold, providing robust evidence for adequate discriminant validity among the measured constructs.

**Table 5:** Correlation analysis results of constructs.

Constructs	1	2	3	4	5	6	7
1. Informativeness	.742						
2. Entertainment	.508**	.728					
3. Interactivity	.488**	.472**	.786				
4. Authenticity	.533**	.520**	.561**	.755			
5. Brand attitude	.421**	.418**	.512**	.477**	.758		
6. Brand loyalty	.457**	.390**	.424**	.454**	.372**	.825	
7. Purchase intention	.655**	.592**	.665**	.666**	.647**	.611**	.805

** *p* < 0.01.

**Table 6:** HTMT (Heterotrait-Monotrait) ratio analysis results of constructs.

Constructs	Interactivity	Interactivity	Authenticity	Brand loyalty	Brand attitude	Entertainment
Interactivity						
Informativeness	0.548					
Authenticity	0.627	0.605				
Brand loyalty	0.474	0.52	0.515			
Brand attitude	0.588	0.489	0.555	0.433		
Entertainment	0.532	0.581	0.592	0.445	0.488	
Purchase intention	0.761	0.761	0.77	0.709	0.769	0.691

### 3.3 Regression analysis of sports brand content characteristics, brand attitude, and brand loyalty

This study conducted multiple linear regression analyses with purchase intention as the dependent variable, while sports brand content characteristics on TikTok, brand attitude, and brand loyalty were used as independent variables. The regression results are presented in Table 7. Multicollinearity Test: The Variance Inflation Factor (VIF) values range from 1.4 to 1.9, all of which are below the threshold of 5, indicating that multicollinearity is not a concern and can be disregarded; The Tolerance values range from 0.5 to 0.7, all of which are greater than 0.1, further confirming the absence of significant multicollinearity issues and demonstrating the robustness of the model. The overall regression model yielded a statistically significant F-value of 312.932 (*P* < 0.001), indicating that the multiple regression model passed the overall significance test and is statistically meaningful. Brand Attitude (*β* = 0.249) has the greatest impact on purchase intention,

**Table 7:** Multiple linear regression analysis.

Variables	Coefficient	Standard error	Standardized coefficient	*t*	VIF	Tolerance
Constant	−.805	.127	–	−6.355		
informativeness	.195	.018	.199	11.069***	1.765	0.566
Entertainment	.134	.020	.115	6.579***	1.659	0.603
Interactivity	.202	.019	.200	10.608***	1.944	0.514
Authenticity	.172	.020	.162	8.685***	1.904	0.525
Brand attitude	.298	.020	.249	14.801***	1.538	0.65
Brand loyalty	.247	.018	.228	13.846***	1.473	0.679

*** *p* < 0.001.

Brand Loyalty (*β* = 0.228) follows as the second strongest predictor, Interactivity (*β* = 0.200) ranks third, Informativeness (*β* = 0.199) and Authenticity (*β* = 0.162) also play positive roles, Entertainment (*β* = 0.115), while still statistically significant, has the weakest effect on purchase intention. These findings highlight the pivotal role of brand attitude and brand loyalty in shaping purchase intention, with brand interactivity, informativeness, and authenticity also exerting significant influence. In contrast, entertainment, while relevant, has a comparatively weaker impact, suggesting that consumers place greater emphasis on informational and interactive brand experiences rather than entertainment alone.

### 3.4 Structural equation modeling

The model fit indices were assessed to evaluate the discrepancy between the proposed model and observed data (absolute fit indices) as well as to compare the proposed model with an ideal model (relative fit indices) (36). This study examined several relative fit indices, including the Root Mean Square Error of Approximation (RMSEA), Goodness-of-Fit Index (GFI), Normed Fit Index (NFI), Incremental Fit Index (IFI), Tucker–Lewis Index (TLI), and Comparative Fit Index (CFI). As shown in Table 8, the Chi-square to degrees of freedom ratio (CMIN/DF) is 2.252, which is below the recommended threshold of 5, indicating an acceptable model fit (58). Additionally, all fit indices meet the established research standards: GFI, AGFI, NFI, IFI, TLI, and CFI all exceed 0.90, indicating a strong model fit. RMR (Root Mean Square Residual) is 0.044, which is below the acceptable threshold of 0.08. RMSEA (Root Mean Square Error of Approximation) is 0.03, also below the recommended threshold of 0.08. These results confirm that the model exhibits a satisfactory fit to the data.

**Table 8:** Results of model fit test.

Model fit index	Optimal standard value	Statistical value	Results
CMIN	–	1704.874	–
DF	–	757	–
CMIN/DF	<5	2.252	Standard
RMR	<0.08	0.044	Standard
GFI	>0.8	0.944	Standard
AGFI	>0.8	0.9336	Standard
NFI	>0.9	0.948	Standard
IFI	>0.9	0.971	Standard
TLI	>0.9	0.968	Standard
CFI	>0.9	0.971	Standard
RMSEA	<0.08	0.03	Standard

This study utilized second-order confirmatory factor analysis to examine the measurement model of content characteristics, with four first-order factors—informativeness, entertainment, interactivity, and authenticity—loading collectively onto a higher-order “content characteristics” construct. All standardized path coefficients from first- to second-order factors were statistically significant (*p* < 0.001), and the first-order factors demonstrated strong intercorrelations (*R* > 0.6), satisfying the prerequisites for higher-order factor formation. Furthermore, reliability and validity metrics across all hierarchical levels exceeded recommended thresholds, confirming the robustness of this conceptual aggregation (Table 9).

**Table 9:** Second-order and first -order reliability and validity metrics.

Order		CR	AVE	RhoA
Second-order	Brand Content Characteristics	0.925	0.636	0.928
First -order	Informativeness	0.879	0.55	0.878
First-order	Entertainment	0.87	0.531	0.868
First-order	Interactivity	0.907	0.619	0.905
First-order	Authenticity	0.888	0.57	0.887

**Figure 3:**
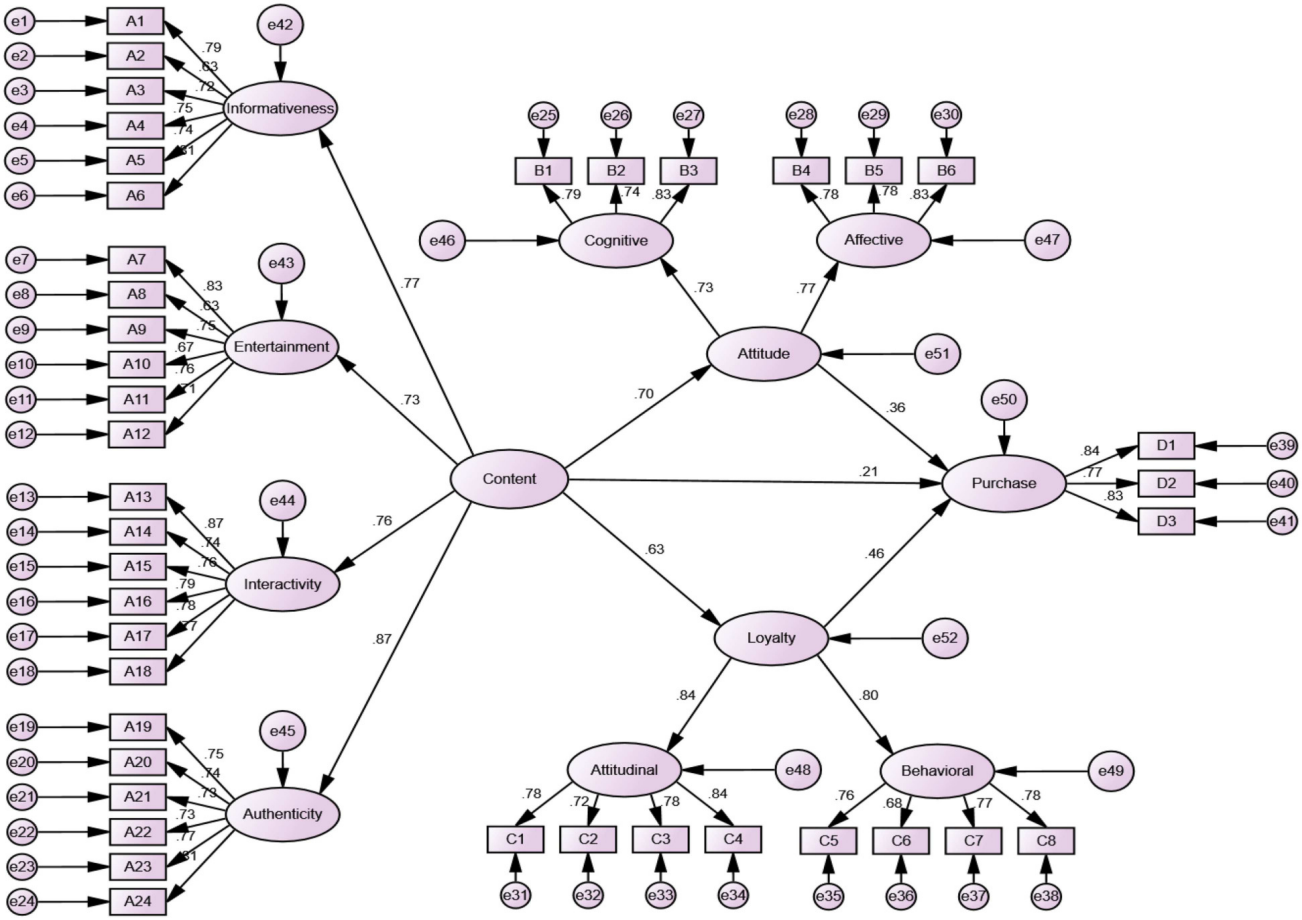
Sem path coefficient model diagram.

The structural equation model (SEM) was utilized to examine the relationships between sports brand content characteristics and consumer behavioral outcomes in the social media context. The standardized factor loadings (ranging from 0.73 to 0.87) indicate strong internal consistency and structural validity. Additionally, the SEM results further confirm that brand content characteristics significantly influence the two mediating variables: brand attitude and brand loyalty. The path coefficients from brand content characteristics to brand attitude (*β* = 0.70) and brand loyalty (*β* = 0.63) are both statistically significant, suggesting that high-quality brand content plays a crucial role in shaping consumer attitudes and fostering brand loyalty. Moreover, brand attitude (*β* = 0.36) and brand loyalty (*β* = 0.46) significantly influence purchase intention, demonstrating their mediating roles in the relationship between brand content characteristics and purchase intention (Figure 3). The findings validate that well-structured and engaging brand content on social media platforms such as TikTok positively impacts consumer attitudes and loyalty, which in turn enhance purchase intention. The strong model fit and significant path coefficients further support the robustness of the proposed research framework.

### 3.5 The mediating role of brand attitudes and brand loyalty, sports brand content characteristics and purchase intentions

In the proposed research model, informativeness, entertainment, interactivity, authenticity serve as independent variables, while brand attitude and brand loyalty function as mediators, and purchase intention is the dependent variable. To further examine the path relationships, this study employed Bootstrapping to validate mediation effects (Table 10). Total Effects: Informativeness→Purchase Intention (Total Effect = 0.32), Entertainment→Purchase Intention (Total Effect = 0.177), Interactivity→Purchase Intention (Total Effect = 0.319), Authenticity →Purchase Intention (Total Effect = 0.275), These findings confirm the existence of significant total effects, indicating that each content characteristic influences purchase intention. Direct Effects: Informativeness→Purchase Intention (Direct Effect = 0.187), Entertainment→Purchase Intention (Direct Effect = 0.077), Interactivity→Purchase Intention (Direct Effect = 0.106), Authenticity→Purchase Intention (Direct Effect = 0.091), Since direct effects are present, it suggests that brand content characteristics exert a direct impact on purchase intention. Indirect Effects (Mediation through Brand Attitude and Brand Loyalty), Informativeness→Brand Attitude→Purchase Intention (Indirect Effect = 0.051), Informativeness→Brand Loyalty→Purchase Intention (Indirect Effect = 0.082), Entertainment→Brand Attitude→Purchase Intention (Indirect Effect = 0.065), Entertainment→Brand Loyalty→Purchase Intention (Indirect Effect = 0.035), Interactivity→ Brand Attitude→Purchase Intention (Indirect Effect = 0.16), Interactivity→Brand Loyalty→ Purchase Intention (Indirect Effect = 0.053), Authenticity→Brand Attitude→Purchase Intention (Indirect Effect = 0.11), Authenticity→Brand Loyalty→Purchase Intention (Indirect Effect = 0.073). The path analysis reveals distinct mechanistic pathways through which content characteristics influence purchase intention. Informativeness demonstrates the strongest total effect (*β* = 0.320), with brand loyalty mediation (*β* = 0.082) representing its predominant pathway. This indicates that providing accurate, authoritative product information primarily creates value through cultivating long-term brand loyalty—the fundamental driver of repeat purchases and stable revenue streams. Interactivity exhibits comparable total effect magnitude (*β* = 0.319) but operates principally through brand attitude mediation (*β* = 0.160). This suggests that interactive features (e.g., liking, commenting, voting) most effectively enhance immediate brand affinity and emotional connection. Entertainment demonstrates the weakest overall influence (*β* = 0.177), with limited direct (*β* = 0.077) and indirect effects, supporting the proposition that mere entertainment possesses limited efficacy in driving purchase behavior. Authenticity emerges as substantively significant (*β* = 0.275), functioning through both attitude and loyalty pathways. This establishes content credibility as the foundational prerequisite for brand trust—without which other content characteristics demonstrate diminished effectiveness. From a psychological perspective, direct effects represent relatively superficial influences, such as immediate interest sparked by content itself. When indirect effects substantially exceed direct effects, this indicates that content's primary impact operates through the cultivation of profound and stable internal psychological constructs. The core value of Douyin content marketing lies not in direct persuasion to purchase, but rather in establishing enduring emotional connections (brand attitude) and reliable behavioral relationships (brand loyalty) through systematic content operations. These cultivated psychological constructs constitute the fundamental drivers of consumer behavior. Regarding brand equity implications, brand attitude and brand loyalty represent core components of brand asset architecture. The dominance of indirect effects demonstrates that high-quality content characteristics are primarily invested in brand equity construction, subsequently leveraging this strategic asset as a powerful fulcrum to activate purchase intention. For new product launches and brand campaigns, priority should be given to developing high-interactivity content initiatives—such as challenge campaigns and live Q&A sessions—to rapidly diminish psychological distance with consumers and enhance brand perception. While entertainment elements may be strategically integrated with informational and interactive components (e.g., through light-hearted product review videos), the core value proposition must remain firmly anchored in substantive information exchange or meaningful interaction. Brands must categorically avoid misleading claims and excessive digital enhancement. Instead, actively encourage authentic user-generated content that reflects genuine product experiences, including balanced presentations of product attributes. Proactive engagement with critical feedback represents a crucial authenticity-preserving behavior that ultimately converts into sustainable brand equity over extended temporal horizons. These results confirm the presence of significant mediation effects, indicating that brand attitude and brand loyalty act as mediators in the relationship between TikTok sports brand content characteristics and purchase intention.

**Table 10:** Mediating effects of brand attitude and brand loyalty on purchase intentions.

Path relationship	Standardized effect size	Bias-corrected		Percentile	
		95%CI		95%CI	
		Lower	Upper	Lower	Upper
Informativeness→Total effect of purchase intention	0.32	0.696	0.801	0.696	0.801
Informativeness→purchase intention direct effect	0.187	0.067	0.32	0.064	0.319
Informativeness→Brand Attitude→Purchase Intention	0.051	0.172	0.378	0.171	0.377
Informativeness→Brand Loyalty→Purchase Intention	0.082	0.237	0.349	0.236	0.347
Entertainment→Total effect of purchase intention	0.177	0.696	0.801	0.696	0.801
Entertainment→purchase intention direct effect	0.077	0.067	0.32	0.064	0.319
Entertainment→Brand Attitude→Purchase Intention	0.065	0.172	0.378	0.171	0.377
Entertainment→Brand Loyalty→Purchase Intention	0.035	0.237	0.349	0.236	0.347
Interactivity→Total effect of purchase intention	0.319	0.696	0.801	0.696	0.801
Interactivity→Purchase intention direct effect	0.106	0.067	0.32	0.064	0.319
Interactivity→Brand Attitude→Purchase Intention	0.16	0.172	0.378	0.171	0.377
Interactivity→Brand Loyalty→Purchase Intention	0.053	0.237	0.349	0.236	0.347
Authenticity→Total effect of purchase intention	0.275	0.696	0.801	0.696	0.801
Authenticity→Purchase intention direct effect	0.091	0.067	0.32	0.064	0.319
Authenticity→Brand Attitude→Purchase Intention	0.11	0.172	0.378	0.171	0.377
Authenticity→Brand Loyalty→Purchase Intention	0.073	0.237	0.349	0.236	0.347

## 4 Discussion

### 4.1 Characteristics of sports brand content on TikTok and purchase intention

The impact of social media advertising value on consumer purchase intention has been extensively studied. Information quality, entertainment value, and authenticity are considered key factors influencing consumers' perceived advertising value and purchase intention (37), thereby validating Hypothesis 1. Interactivity (*β* = 0.200) and Informativeness (*β* = 0.199) are virtually identical. This pattern is further corroborated by the total effects analysis, which shows that informativeness (0.320) and interactivity (0.319) exert nearly equivalent overall influence on purchase intention. Therefore, it can be concluded that these two content characteristics have comparable importance in shaping consumers' purchase decisions, rather than one being distinctly stronger than the other. As the core of brand content, informativeness provides consumers with fundamental brand knowledge. High-quality information enhances brand authenticity, fosters a positive brand attitude, and reduces uncertainty in purchase decisions (38). Meanwhile, authenticity ensures the reliability of brand content, alleviates consumer concerns, and subsequently increases purchase intention (39). Interactivity is crucial in enhancing brand appeal and consumer engagement. Consumers interact with brands on social media through comments, likes, and shares, deepening brand recognition and strengthening both purchase intention and brand loyalty (40). Additionally, entertainment value enhances the attractiveness of brand content and fosters emotional resonance, indirectly influencing consumer purchase decisions (41). Although some studies suggest that entertainment value has a weaker direct impact on purchase intention, it plays a significant role in improving brand recall and shaping brand attitudes, ultimately facilitating consumer purchasing behavior (42). In summary, the characteristics of sports brand content on TikTok influence consumer purchase intention in multiple dimensions. Informativeness strengthens brand authenticity, authenticity fosters consumer trust, interactivity bridges the gap between brands and consumers, and entertainment value enhances brand appeal. Sports brands should develop integrated marketing strategies that combine high-quality information, authentic and transparent content, engaging interactive experiences, and appropriate entertainment elements to enhance consumer brand identification and purchase intention (43). Through precise content marketing, sports brands can establish a solid consumer base in the highly competitive social media landscape and achieve long-term brand growth.

### 4.2 The mediating role of brand attitude in the relationship between TikTok sports brand content characteristics and purchase intention

In the context of social media marketing, brand content characteristics have become critical factors influencing consumer brand attitudes (23). Specifically, previous studies have confirmed that informativeness, entertainment value, interactivity, and authenticity significantly impact brand attitude (44). Moreover, brand attitude plays a crucial role in consumer purchasing decisions. According to the Theory of Reasoned Action (TRA), attitude represents a psychological tendency that effectively predicts an individual's behavioral intention. From the perspective of consumer psychological mechanisms, brand content characteristics influence purchase intention through brand attitude, as a positive brand attitude directly enhances consumer willingness to purchase (45). Additionally, when analyzing the stability and persistence of brand attitude, research has found that consumers' positive evaluations of a brand tend to be highly stable over time and continuously guide their subsequent purchasing decisions (46). Furthermore, the Theory of Planned Behavior (TPB) also underscores the central role of attitude in consumer behavior decision-making. In their application of TPB to analyze consumer brand decision-making mechanisms, Tiwari, Kumar, Kant, and Jaiswal found a significant predictive relationship between brand attitude and purchase intention, indicating that the more positive the attitude, the stronger and clearer the purchase intention (47). Brand content disseminated on social media—its informativeness, entertainment value, interactivity, and authenticity—stimulates consumer cognitive and emotional responses, leading to a positive brand attitude, which significantly enhances purchase intention (23). Similarly, studies on consumer perceptions of social media content have found that a favorable brand attitude not only strengthens brand recognition but also significantly drives actual purchasing behavior (48). Additionally, cross-cultural studies have demonstrated that the impact of brand attitude on purchase intention is stable and widely applicable across different consumer contexts (49), thus validating Hypothesis 2. Based on the Cognition-Affect-Behavior (CAB) model, this study proposes and verifies that brand attitude fully mediates the relationship between brand content characteristics and consumer purchase intention. This conclusion not only aligns with the theoretical expectations of TRA and TPB regarding attitude formation but also expands the existing literature on the relationship between brand content and consumer behavior. It further underscores the central role of brand attitude in social media marketing practices. Therefore, when marketing on the TikTok platform, sports brands should prioritize optimizing content characteristics by enhancing informativeness, entertainment value, interactivity, and authenticity. By doing so, they can continuously reinforce brand attitude, ultimately increasing consumer purchase intention, gaining a competitive market advantage, and driving brand growth.

### 4.3 The mediating role of brand loyalty in the relationship between TikTok sports brand content characteristics and purchase intention

Brand loyalty reflects consumers' long-term commitment and sustained preference for a brand, typically manifested in emotional attachment, trust development, and a stable tendency toward repeat purchases (50). For instance, user-generated content (UGC), characterized by its high authenticity and interactivity, fosters deeper connections with consumers and significantly enhances brand loyalty (51). Likewise, active interactions between brands and consumers can strengthen brand affinity, prompting deeper cognitive, emotional, and behavioral resonance, thereby stabilizing and reinforcing brand loyalty (52). Specifically, high consumer brand engagement directly contributes to the formation of brand loyalty, indicating that interactivity plays a crucial role in building long-term brand relationships (53). In contrast, the role of entertainment value in brand loyalty is more complex. While some studies suggest that entertainment has a limited direct impact on brand loyalty (54), others argue that in certain contexts, entertaining brand content can enhance brand experience, shape a more approachable brand image, and indirectly foster brand loyalty (55). Empirical research widely supports the positive and significant influence of brand loyalty on consumer purchase intention, spanning various industries, including cosmetics, hotel brands, and restaurant services. Furthermore, scholars have systematically analyzed the relationship between brand engagement, brand loyalty, and overall brand equity, highlighting that brand loyalty acts as a sequential mediator between brand engagement and purchase intention (56). Similarly, in the airline service industry, brand heritage has been shown to enhance consumer purchase intention through the mediating roles of brand trust and brand loyalty (57), thereby validating Hypothesis 3. Additionally, brand loyalty effectively reduces the likelihood of consumer brand switching, helping brands maintain a sustainable competitive advantage in highly competitive markets. Based on these findings, this study, through an analysis of TikTok sports brand content marketing cases, further confirms the mediating role of brand loyalty in the relationship between brand content characteristics and consumer purchase intention. These results suggest that sports brands, when formulating social media marketing strategies, should pay particular attention to optimizing content characteristics. Specifically, brands should enhance informativeness, authenticity, and interactivity while strategically incorporating entertainment value to effectively cultivate and reinforce consumer brand loyalty, ultimately leading to higher purchase intention.

## 5 Conclusions

This study analyzes the impact of TikTok sports brand content characteristics on purchase intention and presents the following conclusions based on hypothesis testing: First, TikTok sports brand content characteristics have a direct impact on consumer purchase intention, with informativeness and interactivity being the primary driving force. Second, Brand attitude plays a significant mediating role between sports brand content characteristics and purchase intention. Consumers develop brand attitudes based on content characteristics, which in turn influence their purchase decisions. This finding that brand attitude is a crucial element in content marketing strategies. Last, Brand loyalty serves as a key mediator in the relationship between sports brand content characteristics and purchase intention. Consumers' long-term trust and commitment to a brand further reinforce the impact of content characteristics on purchase intention. The findings of this study validate the central role of social media content characteristics in brand marketing, particularly on TikTok's short-video platform, where interactivity and informativeness are proven to be key factors influencing consumer brand attitude and loyalty. Interactivity enhances brand affinity, fostering active consumer participation, while informativeness strengthens brand professionalism, reducing decision-making risks. Furthermore, the mediating roles of brand attitude and brand loyalty suggest that sports brands must prioritize brand recognition and relationship-building in their content marketing strategies to effectively enhance consumer purchase intention.

## 6 Research limitations and future research directions

Despite the theoretical and practical contributions of this study, several limitations should be acknowledged, which also present opportunities for future research. First, the non-probability sampling method employed—specifically, purposive sampling of consumers from major economically developed cities in China (Beijing, Shanghai, Nanjing, Jinan)—inherently restricts the generalizability of the findings. While this approach was effective for efficiently targeting the study's population of interest, it introduces a potential urban bias. The sample may not adequately represent the behaviors and perceptions of TikTok consumers in less developed cities, rural areas, or regions with different socioeconomic profiles. Consequently, the results are more indicative of trends within affluent, urban contexts rather than being fully representative of the entire population of TikTok sports product buyers in Mainland China. Future studies should strive to employ stratified random sampling techniques across diverse geographic and socioeconomic strata to enhance the external validity and robustness of the findings. Second, the cross-sectional nature of the data limits our ability to infer causal relationships among the constructs. The data were collected at a single point in time, capturing attitudes and intentions as snapshots. While the proposed theoretical model suggests certain directional influences, longitudinal or experimental research designs are required to establish causality and examine the dynamic evolution of brand attitudes and loyalty over time. Finally, although the study focused on key constructs, the model does not encompass all potential variables that could influence consumer behavior on TikTok. Factors such as the specific type of content (e.g., live streams vs. short videos), algorithmic personalization, or the role of social influence from non-brand accounts (e.g., influencers, friends) were not examined. Incorporating these variables in future research could provide a more comprehensive understanding of the drivers of purchase intention on short-video platforms. This investigation primarily examined the direct pathways between content characteristics and psychological mechanisms, while acknowledging the intentional exclusion of significant moderating variables. Specifically, consumer-based factors including brand familiarity and individual personality traits may systematically moderate the efficacy of content characteristics. Subsequent research should incorporate these moderators into expanded analytical frameworks to precisely delineate boundary conditions governing TikTok content marketing effectiveness. Such methodological advancements would enable more nuanced understanding of contextual influences on observed effects. The operational definition of “brand content” in this study was deliberately circumscribed to videos originating from brand-owned channels and authorized co-creators. This conceptual boundary necessarily excluded several ecologically significant components of the contemporary social media landscape, including: organic user-generated content, influencer partnership campaigns, and algorithmically amplified content distribution. Consequently, the generalizability of our findings across the full spectrum of platform dynamics requires prudent qualification. Future investigations should embrace a more inclusive content taxonomy to enhance ecological validity and platform-wide applicability. An additional methodological limitation concerns the omission of consumers' prior brand usage experience and overall platform engagement frequency as statistical controls. These pre-existing behavioral determinants may exert direct effects on purchase intention independent of content characteristics. The failure to partial out these influences introduces potential confounding in estimating the net effects of content marketing variables. Future research designs should incorporate these critical control measures at the questionnaire development phase to facilitate more precise isolation and quantification of content strategy impacts. Based on the empirical evidence, sports brand managers operating on TikTok should implement the following resource optimization strategy: Based on empirical evidence, sports brand managers on TikTok should prioritize budgetary allocation toward high-informativeness and high-interactivity content while significantly reducing resources for purely entertainment-focused formats. A systematic content review mechanism should be implemented to prioritize substantive, engagement-driven content. Creative standards must eliminate superficial product displays and information-deficient messaging, discontinue unidirectional broadcasting and non-reciprocal interactions, and focus on long-term brand equity building rather than immediate conversion metrics. This optimized framework aligns resource distribution with measured effect sizes while sustaining brand-building as the primary strategic objective.

## Data Availability

The raw data supporting the conclusions of this article will be made available by the authors, without undue reservation.
